# Evidence of a trans-kingdom plant disease complex between a fungus and plant-parasitic nematodes

**DOI:** 10.1371/journal.pone.0211508

**Published:** 2019-02-13

**Authors:** David Linnard Wheeler, Jeness Scott, Jeremiah Kam Sung Dung, Dennis Allen Johnson

**Affiliations:** 1 Department of Plant Pathology, Washington State University, Pullman, WA, United States of America; 2 Department of Botany and Plant Pathology, Oregon State University, Madras, OR, United States of America; Tallinn University of Technology, ESTONIA

## Abstract

Disease prediction tools improve management efforts for many plant diseases. Prediction and downstream prevention demand information about disease etiology, which can be complicated for some diseases, like those caused by soilborne microorganisms. Fortunately, the availability of machine learning methods has enabled researchers to elucidate complex relationships between hosts and pathogens without invoking difficult-to-satisfy assumptions. The etiology of a destructive plant disease, Verticillium wilt of mint, caused by the fungus *Verticillium dahliae* was reevaluated with several supervised machine learning methods. Specifically, the objective of this research was to identify drivers of wilt in commercial mint fields, describe the relationships between these drivers, and predict wilt. Soil samples were collected from commercial mint fields. Wilt foci, *V*. *dahliae*, and plant-parasitic nematodes that can exacerbate wilt were quantified. Multiple linear regression, a generalized additive model, random forest, and an artificial neural network were fit to the data, validated with 10-fold cross-validation, and measures of explanatory and predictive performance were compared. All models selected nematodes within the genus *Pratylenchus* as the most important predictor of wilt. The fungus after which this disease is named, *V*. *dahliae*, was the fourth most important predictor of wilt, after crop age and cultivar. All models explained around 50% of the total variation (*R*^*2*^ ≤ 0.46), and exhibited comparable predictive error (RMSE ≤ 1.21). Collectively, these models revealed that the quantitative relationships between two pathogens, mint cultivars and age are required to explain wilt. The ascendance of *Pratylenchus* spp. in predicting symptoms of a disease assumed to primarily be caused by *V*. *dahliae* exposes the underestimated contribution of these nematodes to wilt. This research provides a foundation on which predictive forecasting tools can be developed for mint growers and reminds us of the lessons that can be learned by revisiting assumptions about disease etiology.

## Introduction

Diseases of plants and animals jeopardize food security, public health, and biodiversity [1, 2]. Prevention of disease epidemics requires information about disease monitoring and surveillance [3], biosecurity [4], and prediction of disease emergence [5]. “Prediction”, however, “is difficult, especially about the future” [6, 7]. The accuracy of predictions is limited by the underlying predictability of the system and recognition of all possible outcomes [8]. The availability, use, and interpretation of data as well as the analytical methods used to detect patterns therein also constrain accurate predictions [8]. Further, for infectious diseases, prediction efforts are predicated upon identification of causes of a disease and the conditions that favor outbreaks.

Operationally, disease etiology is elucidated with an inductive and pathogen-centric paradigm, where the organism that causes a disease is identified with Koch’s postulates and thereafter is assumed to be responsible for all symptoms associated with and typified by the original host [9]. Conversely, when the etiology of a disease is complex and evades this reductionist approach [10], a deductive, association-oriented, strategy may be employed to identify candidate biotic and or abiotic factors associated with disease symptoms [11]. Hypothetically, these approaches should converge on the same causal organism(s). In practice, however, the road to causation can be circuitous.

Early dying of potato plants, for example, is a disease with complex etiology that can reduce yields by up to 50% [12]. Despite the potential for devastating losses, symptoms of this disease can be nearly indistinguishable from normal plant senescence but for the premature stage at which infected plants senesce [12, 13]. The subtle expression of disease symptoms coupled with the multitude of organisms associated with these symptoms stifled the elucidation of the etiology of this disease [13, 14]. After years of international research efforts, it was discovered that early dying is primarily caused by the fungus *Verticillium dahliae* Kleb. but other fungi, bacteria, and nematodes can intensify disease expression in different geographic areas [13, 14]. Identification of the organisms responsible for early dying has enabled targeted management strategies that can limit the intensity of epidemics [15].

With the growing awareness of disease complexes like potato early dying, non-culturable microbes, and microbiomes, Koch’s postulates have become difficult to complete for some diseases [11, 16]. Moreover, disease prediction and management strategies are non-starters without fundamental etiological information. Fortunately, statistical and machine learning methods that can be used to elucidate complex microbial interactions have become available [17]. The confluence of accumulating evidence that diseases can be caused by more than one organism and the availability of advanced statistical learning tools has enabled researchers to revisit the assumptions of the disease etiology paradigm, namely that one pathogen causes one disease. The precedent for questioning assumptions demonstrates that this is a tried-and-true approach for gathering intelligence [18] and can be especially impactful for plant and animal diseases [19].

Plant diseases caused by soilborne microorganisms are especially apt targets for the application of these methods because they are often caused by bi-, tri-, or multi-variate groups of microbes. Verticillium wilt of mint, for example, can be exacerbated by inter-kingdom interactions between *V*. *dahliae* and root lesion nematode that culminate in disease over the course of years [20, 21]. While both the fungus, *V*. *dahliae*, and the root lesion nematode, *Pratylenchus penetrans* (Cobb, 1917) Filipjev and Schuurmans Stekhoven, 1941, cause disease separately [21, 22] symptoms caused by both organisms together can be especially severe depending on the strains of *V*. *dahliae* present [21], the mint cultivar, and the age of the mint field. While most mint crops are harvested annually or bi-annually for 4–5 years, some crops grown in highly organic alluvial soils can be grown for up to 20 years. Prevention of Verticillium wilt outbreaks in mint production systems therefore requires development of predictive and explanatory models that synthesize important predictors, guide future control efforts, and elucidate the relationships between the factors that contribute to disease outbreaks.

While the importance of and interactions between *V*. *dahliae* and *P*. *penetrans* are understood in controlled laboratory environments, the role of these organisms in driving disease epidemics under fields conditions are not clear. The objective of this research was to reevaluate the disease etiology of Verticillium wilt of mint by identifying the primary drivers of disease. Soil samples were collected from commercial mint fields that varied in location, the cultivar of mint planted, the age of the crop, and the history of wilt. From each sample, *V*. *dahliae* and plant-parasitic nematodes were quantified. The relationships between wilt symptoms and *V*. *dahliae*, plant-parasitic nematodes, crop cultivar, and stand age were evaluated with four different supervised learning methods. These methods all identified *Pratylenchus* spp. as the most important driver of wilt symptoms. To ensure that *Pratylenchus* spp. were imperative to symptom expression and dissociate the effects of these nematodes from *V*. *dahliae* a bioassay was completed in desiccated field soils that were dried such that *V*. *dahliae* could survive, but *Pratylenchus spp*. could not survive. Overt symptoms of wilt were not observed in bioassay plants grown in soil devoid of *Pratylenchus* spp. The importance of reevaluating disease etiology to learn about the conditions under which diseases are expressed is discussed.

## Materials and methods

### Estimation of plant parasites from commercial mint fields

**Fig 1:**
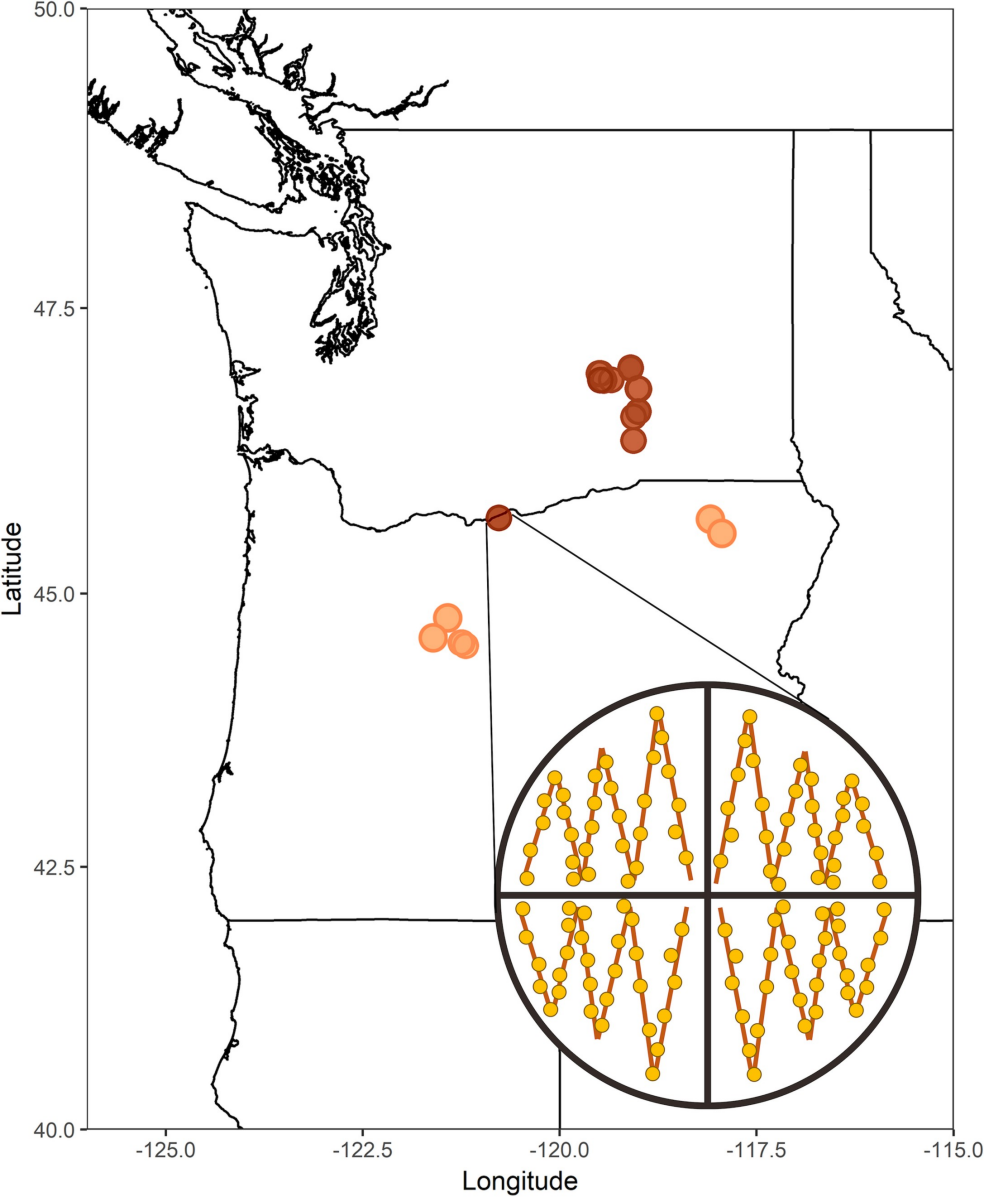
Map of collection sites and sampling scheme. Commercial mint fields in Washington and Oregon states where soil samples were collected and Verticillium wilt symptoms were monitored. Red circles represent fields in Washington while orange circles represent fields in Oregon. The circular inset illustrates the sampling scheme used where lines represent transects and filled circles represent sampling points.

Soil samples were collected between November and February of each sampling year using a hierarchical design where commercial peppermint (*Mentha* x *piperita* L.), native mint (*M*. *spicata* L.), and spearmint (*M*. x *gracilis* Sole.) were selected using a model-based sampling approach to envelop a wide range of pathogen inoculum levels. Sites within fields were selected using a design-based sampling approach to reduce bias within each field. Soil samples were collected from private land with the permission of the land owners. More specifically, a total of 30 fields were sampled in Washington state (n = 17) and Oregon (n = 13) using a stratified random sampling (Fig 1). Three fields in Oregon and two in Washington were sampled consecutively for 2 years and three fields were sampled consecutively for 3 years. Each field was divided into between two to four quarters in order to split fields into approximately equally-sized swaths of land from which samples were collected. From each quarter 30 soil samples were collected with a soil corer along six transects from the top 23 cm of soil. The 30 soil samples from each quarter were subsequently bulked into one composite sample. In total, 96 soil samples were used for further processing. The ages, in years, of the crops from these fields ranged from 1 (n = 48), 2 (n = 25), 3 (n = 17), to 4 (n = 6). The total sample count was not 30 fields x 4 quarters/field = 120 samples because some fields were comprised of only 2 quarters. Soil samples were stored in slightly opened Ziploc bags (SC Johnson, Racine, WI) at 4°C before *V*. *dahliae* and nematode quantification.

From each soil sample *Verticillium* spp. were quantified with two independent methods. Firstly, *Verticillium* spp. were quantified with a culture-dependent assay. Specifically, 0.1 g subsamples of soil were delivered by Anderson air sampler onto 10 plates of semi-selective medium NPX [23] for a total of 1 g [24]. Plates were incubated in darkness for two-weeks at 22°C. Following the incubation, soil was washed from the surface of the plates and *Verticillium-*like colonies were counted.

Secondly, *Verticillium* spp. were quantified with a qPCR assay described by [25]. Genomic DNA was first extracted using the MP FastDNA SPIN Kit for Soil (MP biomedicals, Santa Ana, CA) with several modifications (S1 Protocol and http://dx.doi.org/10.17504/protocols.io.vwie7ce). The master mix for the 20 μl qPCR reaction included 10μl of 2X ABI SYBR Select Mastermix (Applied Biosystems, Foster City, CA), 2μl of each primer (10μM), 2μl of DNA, and 4μl of water. For each sample, three technical replicates were included. To obtain data from samples with potential PCR inhibitors, both non-diluted and 10-fold diluted samples were analyzed. Thermocycler conditions included an initial 10 minute denaturation at 95°C, 40 cycles of denaturation, annealing, and extension at 30s for 95°C, 60°C, and 72°C, where data was collected. Melt curve analysis started at 60°C and increased to 90°C at a rate of 0.3°C/s. Because the primers for the intergenic-spacer region (IGS) used in the qPCR assay above amplify several *Verticillium* spp., we used a [26] multiplex PCR assay to distinguish two *Verticillium* spp. that were similar morphologically.

Nematode extraction, identification and enumeration was competed by Dr. Russ Ingham and Nadine Wade at Oregon State University (Corvallis, OR.). Nematodes were extracted from 250 g soil samples with a modified density centrifugation method [27, 28]. Live nematodes were then identified to genus or species level using morphological features and counted with stereo (40X) and compound (400X) microscopes. Nematode densities were adjusted for soil moisture and are expressed as the number of nematodes/250 g of dry soil.

### Estimation of Verticillium wilt symptoms in commercial mint fields and with a bioassay

Wilt symptoms were quantified in commercial mint fields planted with peppermint, spearmint, and native mint during the growing season after soil samples were collected. While both peppermint and spearmint are susceptible to *V*. *dahliae* native mint is more resistant to *V*. *dahliae* and co-infection with *P*. *penetrans* [21]. Therefore, native mint fields were sampled and plants were included in the bioassay to serve as negative controls. Wilt assessment was completed for each field just before the first and or second harvest for each season. For each field, the number of wilt foci and the number of wilted stems per focus were quantified along the same six transects within each field quarter used for collecting soil samples.

To separate the effects of *V*. *dahliae* and *Pratylenchus* spp. on wilt, symptom development was monitored in mint plants grown in desiccated field soil, where *V*. *dahliae* [24] but not *Pratylenchus* spp. was assumed to survive [29]. Specifically, soil collected from commercial mint fields was homogenized and dried in a greenhouse for 4 weeks at an average temperature of 23.5°C (±11.5°C). Soil was then aerated by incorporation of perlite (Supreme Perlite Co., Portland, OR.) in a 1:1 ratio. Susceptible and resistant mint species, *M*. *gracilis* and *M*. *spicata*, respectively, were subsequently planted in the prepared soil and monitored for disease symptoms. Plants were arranged in a randomized complete block design. Because wilt symptoms accrue over time and may not be expressed within a year, stem sections of mint plants were also assayed for colonization by *V*. *dahliae*. Stems from mint plants were harvested, sectioned into 1-cm pieces, surface disinfested with 0.5% NaOCl for two minutes, dipped in sterilized water, and plated onto NPX medium. Stem sections were incubated at 22°C for 4 weeks and inspected weekly for *Verticillium*-like colonies. *Verticillium* spp. were identified based on morphological features, as described in [30].

### Prediction of Verticillium wilt of mint in commercial mint fields with statistical learning tools

Relationships between wilt symptoms and the abundance of *V*. *dahliae* DNA, *Pratylenchus* spp. counts, the cultivar of mint, and the age of the crop were visualized with locally weighted scatterplot smoothing (lowess) surfaces for continuous predictors and with boxplots for categorical predictors. The smoothness of the lowess surfaces were constrained with smoothing parameters of 0.5. The influence of two predictors on wilt symptoms were visualized with contour plots. Graphs were generated in R (version 3.5.0, R Foundation for Statistical Computing, Austria) with the”ggplot2” [31] and “plotly” packages [32].

The relationship between wilt and the candidate predictors was determined with four supervised learning methods. Estimates of each pathogen were treated as random effects where the mint cultivar and age of the mint crop were treated as fixed effects. Two models with explanatory potential, namely multiple linear regression (MLR) and a generalized additive model (GAM), were used to provide interpretative guidance about the relationships between disease and candidate predictors. To complement these explanatory models, two methods with predictive strength, namely random forests (RF) and artificial neural networks (ANN), were used to advance disease prediction. Our expectation was the RF and ANN algorithms would outperform the MLR and GAM models in predictive performance but that the MLR and GAM would provide interpretable parameter estimates.

All four models were fit and 10-fold cross-validated. The importance of each explanatory variable was quantified using the ‘varImp’ function within the “caret” package in R. The explanatory performance of each model was assessed and compared across all models with adjusted R^2^. Predictive performance was assessed and compared with the root mean squared error (RMSE), repeated 10-fold cross validation, and by comparison of predicted and observed data. Explanatory and predictive performance were compared across all supervised learning methods with tests derived from [33] and [34]. *P*–values were adjusted with a Bonferroni correction. All analyses were completed in R with the “caret” package [35].

The MLR model was used to provide interpretable parameter coefficients to explain relationships between wilt and explanatory variables. Exploratory data analyses and diagnostics were completed to ensure that the assumptions of normality, homoscedasticity, linearity, and orthogonality were satisfied before fitting the MLR model. Putative predictors and interaction terms were then selected with best subset model selection. Finally, the MLR model was fit with the “caret” package in R [35].

The GAM was used to provide flexible, non-linear, relationships between wilt and the explanatory variables. GAMs are extensions of generalized linear models [36] where the response variable is related to smoothed functions of the predictors with a link function. GAM was fitted with the ‘gam’ method within the “caret” package in R. Regression splines were used for smoothing terms. Smoothing parameters were estimated with the Generalized Cross Validation criterion.

The RF algorithm was used to complement the explanatory capacity of the MLR and GAM models with predictive capability. RF is an ensemble method that uses random subsets of predictors to build hundreds to thousands of decision trees over which estimates are averaged to yield predictions for regression problems or modes for classification problems [37]. Hyperparameters were tuned over parameter space with a grid search. A total of 1,000 trees were used to initiate the RF algorithm with the ‘rf’ method within the caret “package” in R.

The ANN algorithm was used, like the RF algorithm, to supplement the explanatory abilities of the MLR and GAM models with predictive power. ANN are algorithmic analogs of biological neural networks. ANN are comprised of nodes that transform inputs, in the form of predictors, to predictive outputs after transmission through a series of hidden nodal layers. Hidden layers combine inputs in weights proportional to their strength with transfer functions that ultimately yield outputs [38]. Hyperparameter tuning for the number of units in the hidden layers was set between 1 to 10 and the weight decays were set from 0.1 to 1. The model averaged neural network algorithm was iterated 5,000 times with the ‘avNNet’ method in the “caret” package in R.

## Results

### Estimation of plant parasites from commercial mint fields

**Fig 2:**
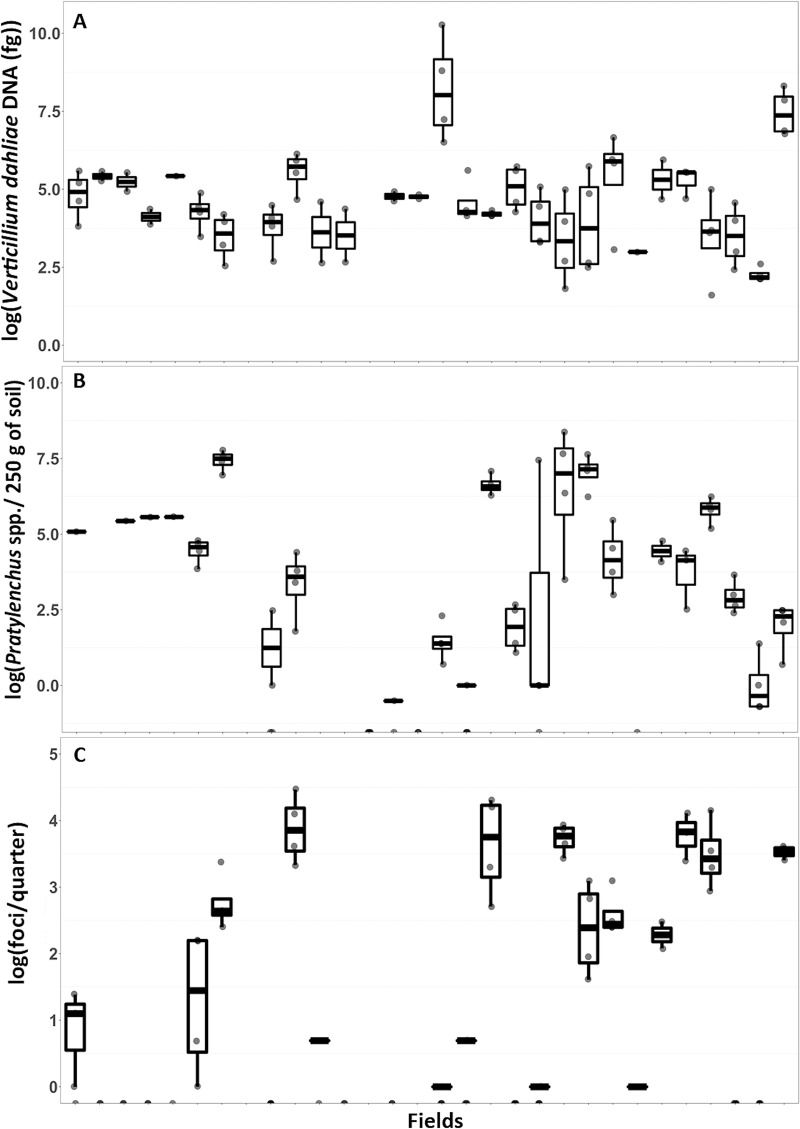
Wilt symptoms and pathogen abundance. Boxplots (minimum, first quartile, median, third quartile, and maximum) of (A) *Verticillium dahliae* inoculum, (B) counts of *Pratylenchus* spp. nematodes, (C) and Verticillium wilt symptoms quantified from commercial mint fields in Washington and Oregon states.

*V*. *dahliae* was detected from all 30 fields (Fig 2A); *V*. *longisporum* was not detected from any fields sampled. Estimates of *V*. *dahliae* DNA are presented because estimates of inoculum are correlated with DNA estimates (r = 0.60, *P* < 0.0001) and the qPCR method was more sensitive than the plating method (S1 Fig). Estimates of *V*. *dahliae* varied across commercial fields sampled in Washington and Oregon from 0 to 135 colony-forming units /g of soil and from 5 to 28,800 femtograms (fg) of DNA (Fig 2). This range of inoculum levels enabled prediction over several orders of magnitude without extrapolation.

Both plant-parasitic and non-parasitic nematodes were detected in the fields sampled. Estimates of all nematodes varied across fields (Fig 2B and S2 Fig). Of the plant-parasitic nematodes detected *P*. *penetrans* [39], *P*. *neglectus* [40], *P*. *thornei* [41], *Paratylenchus* sp. [42], *Criconemella* sp. [39], and *Meloidogyne hapla* [43] are parasitic towards mint species. Aside from the *Pratylenchus* spp. most of the nematodes were not detected from most fields (S2 Fig). Counts of *Pratylenchus* spp. ranged from 0 to 4,345 nematodes/250g of soil (Fig 2B).

### Estimation of Verticillium wilt symptoms in commercial mint fields and with a bioassay

Wilt symptoms, expressed as the number of foci/field, were linearly related to the number of wilted stems/focus, therefor only the former is presented henceforth. Wilt symptoms varied across the fields sampled and ranged from 0 to 87 foci/quarter (Fig 2C). Overt wilt symptoms, typified by chlorosis, necrosis, anthocyanescence, leaf asymmetry, wilting, and stunting, were not observed in the bioassays where resistant and susceptible mint cultivars were grown in desiccated field soil. However, *V*. *dahliae* was isolated from the stems of both susceptible and resistant mint cultivars grown in bioassays using soil from 9/30 and 8/30 fields, respectively (S3 Fig).

**Fig 3:**
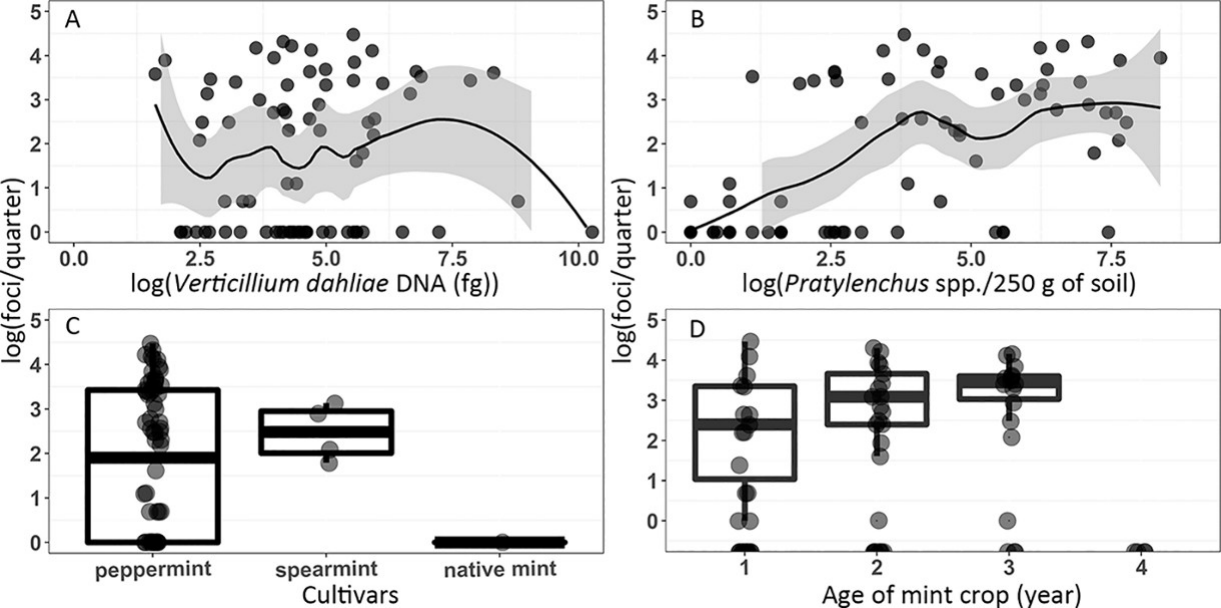
Wilt symptoms as a function of biotic and abiotic predictors. Verticillium wilt symptoms are presented as a function of (A) inoculum of *Verticillium dahliae*, (B) counts of *Pratylenchus* spp., (C) mint cultivars, and (D) age of mint crops.

Wilt symptoms were not linearly related to *V*. *dahliae* inoculum levels (Fig 3A) but were roughly linearly related to *Pratylenchus* spp. counts (Fig 3B). Because linear or non-linear relationships between each organism and wilt symptoms did not appear to explain most of the variation in these data, additional predictors were added before the learning methods were fit and validated. For model building, all *Pratylenchus* spp. were pooled together because *P*. *penetrans*, *P*. *neglectus*, and *P*. *thornei* are all associated with mint crops [39, 40, 41], and prediction with each species alone would require extensive imputation and additional observations.

### Prediction of Verticillium wilt of mint in commercial mint fields with statistical learning tools

**Fig 4:**
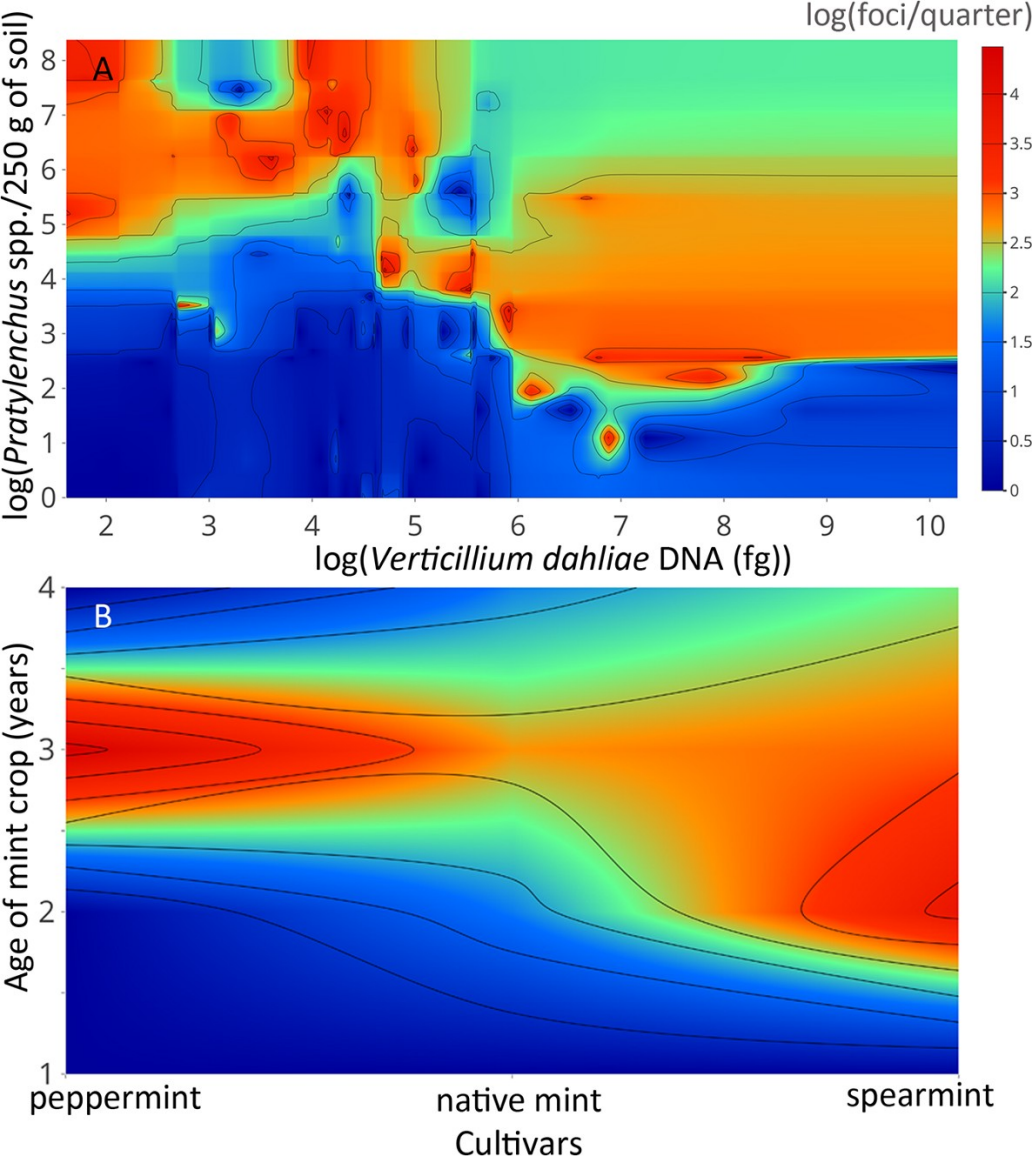
Wilt symptoms as a function of combined biotic and abiotic predictors. Bivariate plots illustrate the relationship between wilt and (A) *Verticillium dahliae* and *Pratylenchus* spp., and (B) mint cultivars and the age of mint crops. Wilt intensity is expressed in color where red represents severe wilt and blue represents no wilt.

Wilt symptoms were not strongly related to *V*. *dahliae* inoculum likely because of several influential observations that pulled the smoothed lowess line away from the 45 degree line expected for a positive correlation (Fig 3). Wilt was positively related to counts of *Pratylenchus* spp. until approximately 50 nematodes were present, after which wilt symptoms asymptote (Fig 3B). The severity of wilt was dependent on the mint cultivar with symptoms being most variable in the peppermint fields, were greatest in the spearmint fields, and lowest in the native mint fields (Fig 3C). Wilt increased with increasing age of the mint crop up until the 4^th^ year, where available data were too sparse to make inferences (Fig 3D). The combined influence of *V*. *dahliae* and *Pratylenchus* spp. on wilt illustrates that disease intensity was greatest under two conditions: when *V*. *dahliae* inoculum was high and *Pratylenchus* spp. counts were low and in the inverse scenario (Fig 4A). The combined influence of mint cultivars and the age of the mint crop on wilt severity illustrates that disease intensity was greatest in 2 to 3 year old spearmint crops and 3 year old peppermint crops (Fig 4B).

**Fig 5:**
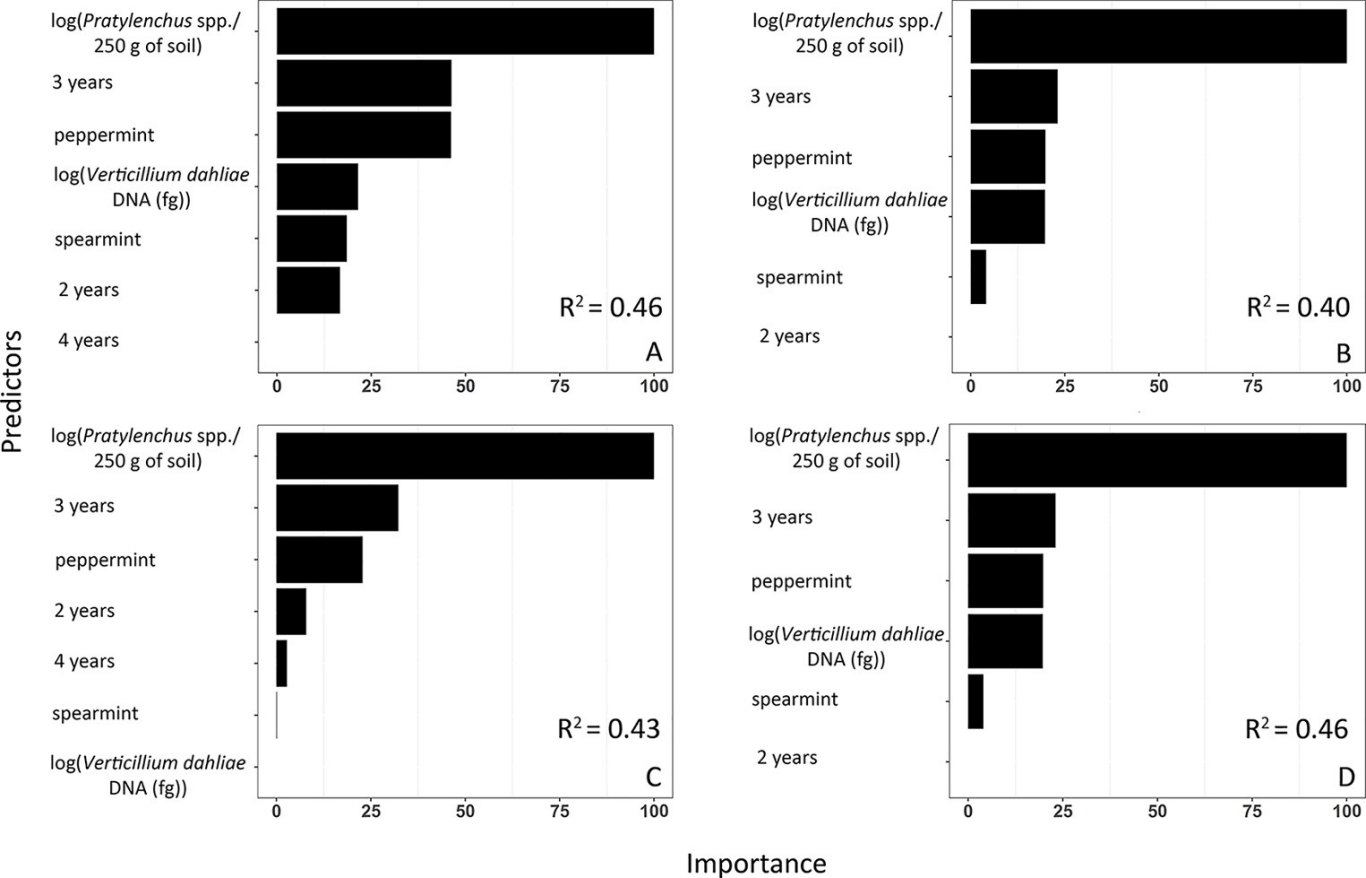
Contribution of each predictor to wilt. Importance of Verticillium wilt predictors and explanatory performance, expressed as coefficients of determination (R^2^), of (A) the multiple linear regression model, (B) generalized additive model, (C) random forest, (D) and artificial neural networks.

The assumptions required to interpret the MLR model were not satisfied, except for orthogonality, which was satisfied by inspection of scatterplot matrices and variance inflation factor scores, which ranged between 0.8 and 1.2. Remedial measures were not pursued to enable comparisons across all supervised learning methods. *V*. *dahliae* inoculum, counts of *Pratylenchus* spp., mint cultivars, and the age of mint crops were selected as predictors with best subsets selection. Coefficients, test statistics, and *P–*values are presented in Table 1. All predictors were positively associated with wilt, except for four year old mint fields which exhibited a slight negative but insignificant relationship with wilt (Table 1). Of the predictors, counts of *Pratylenchus* spp. imparted the strongest effects on wilt severity (Fig 5).

**Table 1:** Effects of predictors on wilt symptoms from the two statistical models.

Predictor	MLR			GAM		
	partial slope	t	*P*-value	edf	F	*P*-value
log(*Verticillium dahliae* DNA (fg))	0.17	1.66	0.03	4.23	3.34	0.008
log(*Pratylenchus* spp. /250 g of soil)	0.44	7.12	<0.0001	8.60	9.77	<0.0001
				estimate	t	
Mint cultivar						
peppermint	2.41	3.41	0.007	1.32	2.71	0.008
spearmint	1.60	1.55	0.13	0.61	1.10	0.32
native spearmint	.	.	.	.	.	.
Field age						
1 year	.	.	.	.	.	.
2 years	0.36	1.33	0.32	0.15	0.22	0.82
3 years	1.08	3.45	0.004	1.11	3.86	0.003
4 years	0.14	-0.24	0.83	.	.	.

Coefficient estimates, test-statistics, the effective degrees of freedom (edf), and *P–*values for predictors of wilt used in multiple linear regression (MLR) and generalized additive models (GAM). Dots represent instances where insufficient data prevented estimation within a row or column.

Like the MLR model, the GAM model, RF, and ANN algorithms identified *Pratylenchus* spp., the age of the mint field, and the cultivar of mint as the most important predictors of wilt (Fig 5). The strength and significance of the coefficients from the GAM model were comparable to the MLR model but differed in form for the continuous predictors. The relationships between wilt and both *V*. *dahliae* and *Pratylenchus* spp. were non-linear, as illustrated by effective degrees of freedom values greater than 1 (Table 1).

**Fig 6:**
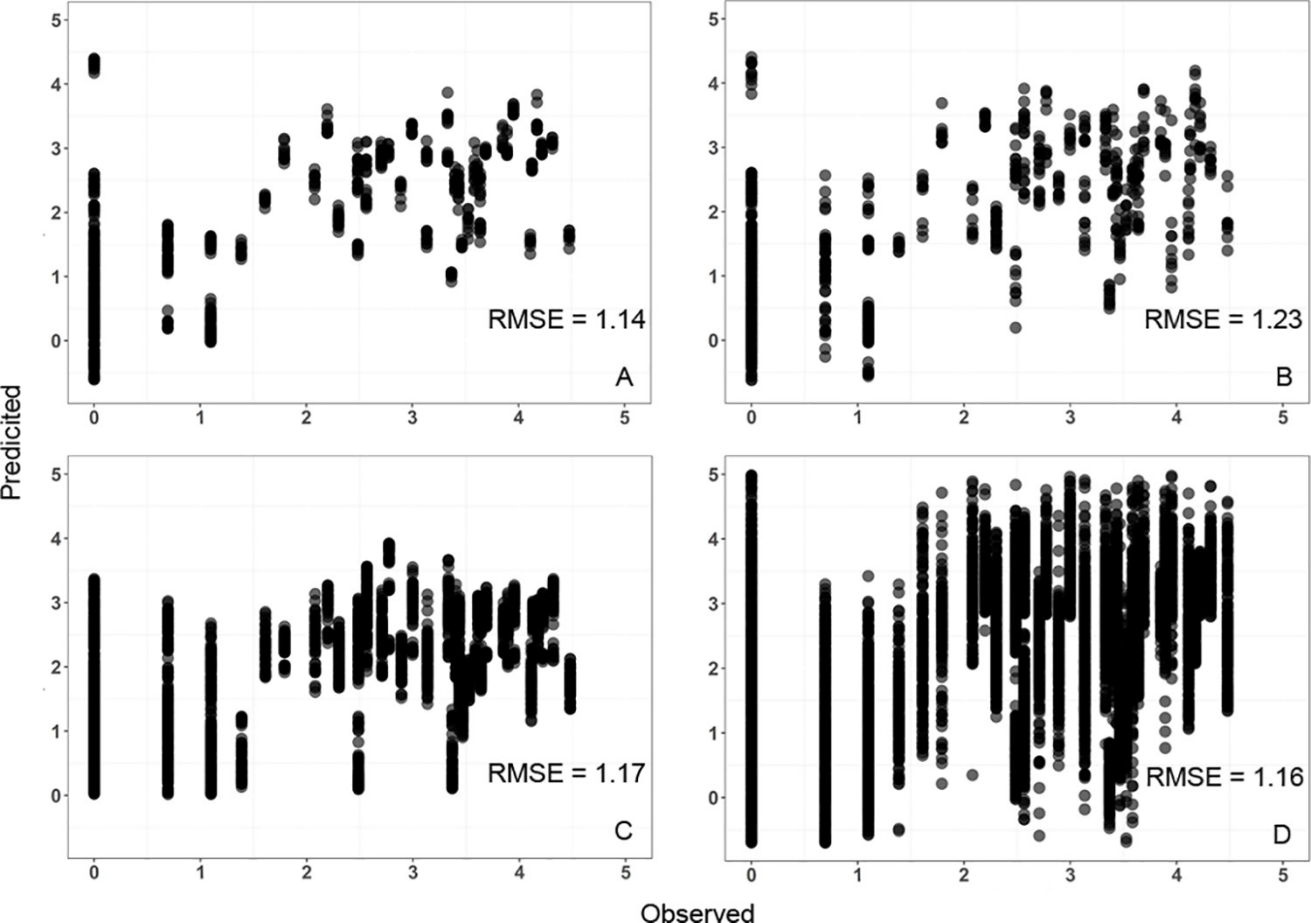
Predictive performance of statistical learning methods. Verticillium wilt symptoms predicted by the supervised learning methods versus observed and predictive performance, expressed as root mean square error (RMSE), of the (A) multiple linear regression model, (B) generalized additive model, (C) random forest, and (D) artificial neural networks.

All supervised learning methods explained approximately 50% of the variation in the data (Fig 5). Adjusted coefficients of determination (R^2^) were not statistically different from each other (*P* ≥ 0.37). Similarly, the predictive performances, expressed as RMSE, were comparable across each learning method and ranged from 1.14 to 1.21 (Fig 6). Further, RMSEs differed only between the GAM and ANN (*P* = 0.03). Finally, all methods predicted wilt when no wilt was observed and underestimated high levels of wilt (Fig 6).

## Discussion

Prevention of disease epidemics depends on a variety of factors, including but not limited to accurate etiological information and predictive tools. The availability of advanced statistical learning tools has enabled researchers to elucidate complex host-pathogen interactions and generate predictive tools to prevent epidemics. Supervised machine learning techniques were used here to describe and predict the relationships between symptoms of Verticillium wilt of mint and candidate predictors. The fungus that causes symptoms typified by Verticillium wilt, *V*. *dahliae*, was a weak predictor of wilt. Plant-parasitic nematodes within the genus *Pratylenchus*, however, were paramount to all other predictors, including mint cultivar, crop age, and *V*. *dahliae* inoculum levels. All of the supervised learning methods used here, from the proverbial MLR to the more modern RF and ANN, explained and predicted wilt comparably. These results underscore the importance of returning to first principles to reevaluate assumptions about disease etiology and ultimately cultivate a comprehensive understanding of disease epidemiology to enable prevention.

The plant pathogenic fungus, *V*. *dahliae*, after which Verticillium wilts are named was consistently identified as the weakest predictor of mint wilt by all supervised learning methods used herein. This discovery might be explained by the presence of a diversity of *V*. *dahliae* strains that vary, phenotypically, in the ability to cause disease on mint [44]. This explanation is corroborated by the occurrence of high levels of wilt, presumably caused by highly aggressive strains, in soils infested with low levels of *V*. *dahliae* inoculum and vice versa (Fig 3). The cultural and sequence-based methods used here to quantify *V*. *dahliae* do not differentiate among strains of this fungus and, therefore, might overestimate effective inoculum, especially if strains that are not pathogenic towards mint are present. Low levels of disease in some mint fields where soils were infested with *V*. *dahliae* might be explained by soils that suppress disease symptoms despite the presence of *V*. *dahliae*. Although there are no documented examples of suppressive soils in the Verticllium wilt of mint pathosystem, suppressive soils are operative in some potato field soils infested with *Verticillium dahliae* [45]. Spatial and temporal variability [46] might have also contributed to the smaller than expected contribution of *V*. *dahliae* to wilt expression. For example, wilt varies across fields and some symptoms may have escaped visual detection because they are expressed at the scale of one leaf in a field comprised of 100 acres. Similarly, wilt varies through time [46] and it is possible that we sampled prior to symptom expression for some of the younger crops. The observation that overt wilt symptoms were not observed in susceptible or resistant bioassay plants, despite low incidences of infected stems, provides evidence that the strains present in the fields sampled were either not pathogenic towards mint or needed more time to incite symptoms.

Plant-parasitic nematodes in the genus *Pratylenchus* were strongly associated with wilt symptoms assumed to be caused by the fungus *V*. *dahliae*. Specifically, *Pratylenchus* spp. were at least twice as important in explaining wilt symptoms as the other predictors, regardless of the learning method used. The importance of *Pratylenchus* spp. in contributing to symptoms of Verticillium wilt might be explained by the similarity of symptoms induced by *V*. *dahliae* and *P*. *penetrans* in mint. For example, both organisms can induce stunting and anthocyanescence in susceptible mint plants [22, 47] but only *V*. *dahliae* causes asymmetrical leaf morphology [47] and only *P*. *penetrans* weakens mint roots [22]. Differentiation of these symptoms, therefore, requires astute inspection and manipulation of each plant. Conversely, the organisms responsible for symptoms can be inferred with a bioassay where one organism is selectively terminated and symptom expression is monitored. The absence of wilt symptoms in bioassay plants that were grown in desiccated field soil where *P*. *penetrans* could not survive [29] provides evidence that *Pratylenchus* spp. were likely more responsible than *V*. *dahliae* for the wilt symptoms observed in commercial fields. This claim is further supported by the observation that some nematicides can delay development of Verticillium wilt symptoms or increase yields in fields infested with both pathogens [48]. These results are impactful for growers, who might reassess their disease management strategies to target nematodes as well as fungi, and scientists, who might reassess their research efforts to envelop both organisms. Finally, although strong evidence of an association between *Pratylenchus* spp. and wilt is presented here, causation-oriented experiments should be completed before major changes to disease management or research programs are implemented.

While *Pratylenchus* spp. and, to a lesser extent, *V*. *dahliae* were needed to explain wilt expression in commercial mint fields, an interaction between these two organisms was not detected, as might be expected. The absence of this interaction might be explained by the presence of *V*. *dahliae* strains that do not interact synergistically with *P*. *penetrans*, native mint plants [21] or, although unprecedented, *P*. *penetrans* strains that do not interact with *V*. *dahliae*. Furthermore, it is possible that only a subset of the *Pratylenchus* spp. pooled together for this research interact synergistically with *V*. *dahliae* [49]. Finally, although a statistically significant interaction was not detected during model selection, it is possible that a weak but biologically significant interaction was operative yet undetected. This conjecture is supported by the severe levels of wilt observed when the abundances of *V*. *dahliae* and *Pratylenchus* spp are inversely related. Contrary to the expectation that wilt would be most severe when both pathogens were most abundant, Fig 4 reveals that wilt intensity is mild under these conditions. One interpretation of this observation is that wilt expression is dependent on the density of both organisms until a threshold is achieved and thereafter, competition and or antagonism ensue. Together, these results remind us that more fundamental research is needed to describe trans-kingdom behavioral relationships between the microorganisms that affect our food supply.

More information about the drivers of disease emergence and expression are also needed to enhance model performance. All of the supervised learning methods used here described approximately 50% of the total variation in the wilt intensity data. Given that these learning methods spanned the interpretability vs. flexibility spectrum described by [37] it seems unlikely that this unexplained variance might be reduced by the application of another model or algorithm. Instead, the addition of more observations and predictors from the disease triangle, such as environmental factors (e.g. soil physical, chemical, and biological properties) and host and pathogen genotypes, might inflate metrics of explanatory and predictive performances. Finally, deployment of faster, more sensitive, and comprehensive disease monitoring and surveillance systems, like unmanned aerial vehicles, would populate large data sets and enable finer resolution predictions.

The research presented here demonstrates the potential for generating new information about disease etiology by reevaluating the vertices of the disease triangle with the application of machine learning techniques. Specifically, this research exposes the potential rewards of exercising open minds and the costs of maintaining the status quo. The rewards here include a more comprehensive and nuanced understanding of the drivers of a plant disease and prediction tools to guide preventative management strategies. The costs of maintaining the assumption that *V*. *dahliae* alone is the main problem for mint growers in the Pacific Northwest of the United States include the allocation of various resources, from research time and capital to soil-fumigants, to a problem that may not warrant these endowments. While it is certainly impractical to apply this approach to every disease, especially those for which efficacious treatments are available, the rewards of possibly preventing epidemics and conserving resources supersede the risks of status quo confirmation.

## Supporting information

S1 Protocol Protocol for genomic DNA extraction and quantitative real-time PCR. (DOCX)

S1 Fig Relationship between assays for *Verticillium dahliae* quantification. Correlations between estimates of *Verticillium dahliae* from the quantitative real-time PCR (qPCR) assay and the traditional culture-dependent method. Samples from Washington state are represented in red while samples from Oregon are represented in orange. The correlation coefficient and *P-*value for all samples are shown in gray while those for samples from Washington and Oregon are shown in red and orange, respectively.
(TIF)

S2 Fig Plant-parasitic nematode counts. Counts of plant-parasitic nematodes, including *Pratylenchus* spp. (a), *Criconemella* sp. (b), *Paratylenchus* spp. (c), and *Meloidogyne* spp. (d) are presented for each field.
(TIF)

S3 Fig Stem infection of bioassay plants. Incidence of susceptible (*Mentha gracilis*) and resistant (*M*. *spicata*) mint stems infected with *Verticillium dahliae* after a season of growth in desiccated soils collected from commercial mint fields.
(TIF)

S1 Dataset Data for model selection, validation, and comparisons. Raw data collected from fields including field locations, mint cultivars, stand age, nematode counts, estimates of *Verticillium dahliae*, the number of wilt foci/ field quarter and wilted stems/ focus, and the incidence of mint stems infected with *V*. *dahliae*.
(CSV)

## References

[1] Pennisi E. Armed and dangerous. Science. 2010;327: 804–805. 10.1126/science.327.5967.804 20150482

[2] Institute of Medicine. Fungal Diseases: An Emerging Threat to Human, Animal, and Plant Health. Washington, DC: The National Academies Press; 2011. 22259817

[3] Holmes EC, Rambaut A, Andersen KG. Pandemics: spend on surveillance, not prediction. Nature. 2018;558: 180–182. 10.1038/d41586-018-05373-w 29880819

[4] Fisher MC, Henk DA, Briggs CJ, Brownstein JS, Madoff LC, McCraw SL. et al Emerging fungal threats to animal, plant and ecosystem health. Nature. 2012;484: 186–194. 10.1038/nature10947 22498624 PMC3821985

[5] Carroll D, Daszak P, Wolfe ND, Gao GF, Morel CM, Morzaria S. et al The global virome project. Science. 2018;359: 872–874. 10.1126/science.aap7463 29472471

[6] Annas GJ. Precatory prediction and mindless mimicry: the case of Mary O’Connor. The Hastings Center Report. 1988;18: 31–33. 10.2307/3563046 3147972

[7] Ulam SM. Adventures of a mathematician. New York: Charles Scribner’s Sons: 1976.

[8] Beckage B, Gross LJ, Kauffman S. The limits to prediction in ecological systems. Ecosphere. 2011; 2, article 125. 10.1890/ES11-00211.1

[9] Koch R. Xth International Congress of Medicine. Berlin; 1890.

[10] Evans AS. Causation and Disease. 1^st^ ed. Boston: Springer; 1993.

[11] Wallace HR. The diagnosis of plant diseases of complex etiology. Ann. Rev. Phytopathol. 1978;16: 379–402.

[12] Powelson ML, Rowe RC. Biology and management of early dying of potatoes. Annu. Rev. Phytopathol. 1993;31: 111–126. 10.1146/annurev.py.31.090193.000551 18643764

[13] Rowe RC, Davis JR, Powelson ML, Rouse DI. Potato Early Dying: causal agents and management. Plant Dis. 1987;71: 482–489.

[14] Martin MJ, Riedel RM, Rowe RC. *Verticillium dahliae* and *Pratylenchus penetrans*: interactions in the early dying complex of potato in Ohio. Phytopathology. 1982;72: 640–644.

[15] Rowe RC, Powelson ML. Potato early dying: management challenges in a challenging production environment. Plant Dis. 2002;86: 1184–1193. 10.1094/PDIS.2002.86.11.1184 30818465

[16] Byrd AL, Segre JA. Adapting Koch’s postulates: criteria for disease causation must take microbial interactions into account. Science. 2016;351: 224–226. 10.1126/science.aad6753 26816362

[17] Lo C, Marculescu R. MPLasso: Inferring microbial association networks using prior microbial knowledge. PLoS Comput. Biol. 2017;13: 12 Available from: https://journals.plos.org/ploscompbiol/article?id=10.1371/journal.pcbi.1005915 10.1371/journal.pcbi.1005915 PMC5760079 29281638

[18] Heuer RJ. Psychology of Intelligence Analysis. 1^st^ ed. Washington. D.C.: Center for the Study of Intelligence; 1999

[19] Sciacca G, Nicoletti A, Lo Fermo S, Mostile G, Giliberto C, Zappia M. Looks can be deceiving: three cases of neurological diseases mimicking Guillain-Barrè syndrome. Neurol. Sci. 2016;37: 541–545. 10.1007/s10072-015-2450-4 26707616

[20] Back MA, Haydock PPJ, Jenkinson P. Disease complexes involving plant parasitic nematodes and soilborne pathogens. Plant Pathol. 2002;51: 683–697. 10.1046/j.1365-3059.2002.00785.x

[21] Johnson DA, Santo GS. Development of wilt in mint in response to two pathotypes of *Verticillium dahliae* and co-infection by *Pratylenchus penetrans*. Plant Dis. 2001;85: 1189–1192. 10.1094/PDIS.2001.85.11.1189 30823166

[22] Ingham RE, Merrifield K. Biology and management of nematodes in mint. IPPC Publication No. 996. Integrated Plant Protection Center. Corvallis: Oregon State University; 1996. pp. 39.

[23] Kabir Z, Bhat RG, Subbarao KV. Comparison of media for recovery of *Verticillium dahliae* from soil. Plant Dis. 2004;88: 49–55. 10.1094/PDIS.2004.88.1.49 30812456

[24] Butterfield EJ, DeVay JE. Reassessment of soil assays for *Verticillium dahliae*. Phytopathology. 1977;67: 1073–1078. 10.1094/Phyto-67-1073

[25] Wei F, Fan R, Dong H-T, Shang W.-J, Xu X-M, Zhu H-Q. Threshold microsclerotial inoculum for cotton Verticillium wilt determined through wet-sieving and real-time quantitative PCR. Phytopathology. 2015;105: 220–229. 10.1094/PHYTO-05-14-0139-R 25098492

[26] Inderbitzin P, Davis RM, Bostock RM, Subbarao KV. Identification and differentiation of *Verticillium* species and *V*. *longisporum* lineages by simplex and multiplex PCR assays. PLoS One. 2013;8: 6: Available from: https://journals.plos.org/plosone/article?id=10.1371/journal.pone.0065990 10.1371/journal.pone.0065990 PMC3688845 23823707

[27] Ingham RE. Nematodes In: Weaver RW, Angle JS, Bottomly PJ, editors. Methods of soil analysis, Part 2, Microbiological and biochemical properties. Madison: Soil Sci. Soc. of Amer.;1994 pp. 459–490.

[28] Jenkins WR. A rapid centrifugal-flotation technique for separating nematodes from soil. Plant Dis. 1964;48: 692.

[29] Kable PF, Mai WF. Influence of soil moisture on *Pratylenchus penetrans*. Nematologica. 1968;14: 101–122.

[30] Inderbitzin P, Bostock RM, David MR, Usami T, Platt HW, Subbarao KV. Phylogenetics and taxonomy of the fungal vascular wilt pathogen *Verticillium*, with the description of five new species. PloS One. 2011;6: 12: Available from: https://journals.plos.org/plosone/article?id=10.1371/journal.pone.0028341 10.1371/journal.pone.0028341 PMC3233568 22174791

[31] Wickham H. ggplot2: Elegant Graphics for Data Analysis. New York: Springer-Verlag; 2016.

[32] Sievert C. plotly for R. 2018. Available from: https://plotly-book.cpsievert.me Cited 7 September 2018

[33] Hothorn T, Leisch F, Zeileis A, Hornik K. The design and analysis of benchmark experiments. Journal of Computation and Graphical Statistics. 2005;14: 675–699. 10.1198/106186005X59630

[34] Eugster MJA, Hothorn T, Leisch F. Exploratory and inferential analysis of benchmark experiments. Tech. Rep. 30. 2008. Department of Statistics, University of Munich.

[35] Kuhn M. Building predictive models in R using the Caret package. Journal of Statistical Software. 2008;28: 1–26. 10.18637/jss.v028.i07 27774042 PMC5074077

[36] Hastie T, Tibshirani R. Generalized Additive Models. Statist. Sci. 1986;1: 297–318. 10.1177/096228029500400302 8548102

[37] James G, Witten D, Hastie T, Tibshirani R. An introduction to statistical learning. Berlin: Springer; 2013.

[38] Jiaconda. A concise history of neural networks. Medium. 2016. Available from: https://medium.com/@Jaconda/a-concise-history-of-neural-networks-2070655d3fec

[39] Merrifield KJ, Ingham RE. Population Dynamics of *Pratylenchus penetrans*, *Paratylenchus* sp. and *Criconemella xenoplax* on western Oregon peppermint. J Nematol. 1996;28: 557–564. 19277174 PMC2619716

[40] Kleynhans D, Van den Berg E, Swart A, Marias M, Buckley N. Plant nematodes in South Africa. South Africa: Agricultural Research Council; 1996.

[41] Ingham RE. OSU Nematode Testing Service- Pratylenchus on Oregon Crops. 1998. Available from: http://plant-clinic.bpp.oregonstate.edu/nematodes-pratylenchus/ Cited 6 September 2018

[42] Faulkner LR. Pathogenicity and population dynamics of *Paratylenchus hamatus* on *Mentha* species. Phytopathology. 1964;54: 344–348.

[43] Eshtiaghi H. Effects of the northern root-knot nematode (*Meloidogyne hapla* Chitwood 1949) on Mitcham peppermint (*Mentha piperita* L.) and Scotch spearmint (*Mentha cardiaca* Baker). Ph.D dissertation, Oregon State University. 1975.

[44] Dung JKS, Peever TL, Johnson DA. *Verticillium dahliae* populations from mint and potato are genetically divergent with predominant haplotypes. Phytopathology. 2013;103: 445–459. 10.1094/PHYTO-06-12-0133-R 23113547

[45] Larkin RP, Honeycutt CW, and Olanya OM. Management of Verticillium wilt of potato with disease-suppressive green manures and as affected by previous cropping history. Plant Dis. 2011: 95:568–576. 30731947 10.1094/PDIS-09-10-0670

[46] Johnson DA, Zhang H, Alldredge JR. Spatial pattern of Verticillium wilt in commercial mint fields. Plant Dis. 2006;90: 789–797. 10.1094/PD-90-0789 30781241

[47] Horner CE. Pathogenicity of *Verticillium* isolates to peppermint. Phytopathology. 1954;44: 239–242.

[48] Schultz OE, Cetas RC. Evaluation of granular nematicides for control of ‘Early Maturity Wilt’ of potatoes in New York State. Proc. Br. Crop Prot. Conference (titled Pest and Diseases). 1977;2: 491–498.

[49] Wheeler TA, Riedel RM. Interactions among *Pratylenchus penetrans*, *P*. *scribneri*, and *Verticillium dahliae* in the Potato Early Dying disease complex. J. Nematol. 1994;2: 228–234. PMC2619486 19279885

