# Supplementary material for: Evidence of a trans-kingdom plant disease complex between a fungus and plant-parasitic nematodes
Source: PLoS One. 2019 Feb 13;14(2):e0211508. doi: 10.1371/journal.pone.0211508 (PMC6373923; doi:10.1371/journal.pone.0211508)
Supplement: S1 Protocol — (DOCX) [file pone.0211508.s001.docx]

**Protocol for DNA Extraction and Quantitative PCR Detection of *Verticillium dahliae* from Soil**

**Pre-Extraction Sample Preparation:**

1. The soil cores are combined, air-dried and processed through a sieve (No. 10/2.0 mM). Dried soil samples are stored in a cool dark place until use. When stored in the fridge, keep them in sealed plastic bags to prevent the soil from absorbing moisture.
2. Place a 5-10 g subsample of soil into a 15 or 50 ml conical polyethylene tube. Cover the tube with a KymWipe to allow moisture and air to move through the sample and keep the sample in the tube. Freeze the subsample of soil at -80°C for at least 3 hours.
3. The frozen subsample is then freeze dried under a vacuum using a lyophilizer until dry. Drying time varies and ranges from 4 to 24 hours, but could be longer depending soil type.

**DNA Extraction**

- - DNA Extraction Kit: MP FastDNA™ SPIN Kit for Soil (MP Biomedicals; cat no.: 116560200; cost is $230-290 for 50 reactions or 25 soil samples).
    - 100 ml of 100% ethanol (*not included in the kit*) needs to be added to the concentrated SEW-M solution prior to use.
    - Twice as much lysing matrix E (MP Biomedicals; cat no.: 116914050) is used as compared to other kit components.

1. Add 500 mg of freeze-dried soil to a lysing matrix E tube (*supplied with the kit*).
   1. *Use two tubes per soil sample so that DNA is extracted from a total of 1 gram of soil per sample.*
2. Pre-grind the samples in a FastPrep-24 5G (MP Biomedicals; cat no.: 116005500 ) for 40 sec at a speed setting of 6.0.
3. Add 828 µl of sodium phosphate buffer (*supplied with the kit*) to each tube.
4. Add 150 µl of nonfat powdered milk suspension (133 mg/ml) to each tube (*this is not supplied with the kit*).
5. Add 122 µl of MT buffer (*supplied with the kit*) to each tube.
6. Homogenize in the FastPrep machine for 40 seconds at a speed setting of 6.
7. Centrifuge at 14,000 X g for 10 min.
8. Transfer supernatant to a clean 2.0 ml microfuge tubes *(these tubes are not supplied with the kit).*
9. Add 250 µl PPS (Protein Precipitation Solution, *supplied with the kit*) and mix by inverting the tube 10 times.
10. Centrifuge at 14,000 X g for 5 minutes to pellet the precipitate.
    1. *When transferring the supernatant to the 1 ml tube containing binding matrix (steps 11-12) avoid transferring any of the precipitate.*
11. Shake the binding matrix suspension to re-suspend and add 1.0 ml to each 15 ml tube.
    1. *The two 0.5 g subsamples will be pooled in step 12, so only one 15 ml tube for each soil sample is needed*.
12. Add the supernatants from step 10 to the 15 ml tube containing binding matrix.
13. Invert by hand for 2 min to allow the DNA to bind to the binding matrix. Place the tubes in a rack for 5 minutes to allow the silica matrix to settle in the tube.
14. Remove and discard 0.75 ml of the supernatant by pipetting. Be very careful not to disturb the silica matrix.
15. Gently re-suspend the binding matrix in the remaining amount of supernatant by pipetting.
16. Transfer 600 µl of the mixture to a spin filter and centrifuge for 1 min at 14,000 X g.
17. Empty the catch tube and add 500 µl more of the re-suspended silica matrix mixture. Repeat steps 15 and 16 until all of the silica matrix mixture has been added to the spin filter.
18. Add 500 µl of prepared SEWS-M and gently re-suspend the pellet using the force of the liquid from the pipet tip.
    1. *Ensure that the ethanol has been added to the SEWS-M before use.*
19. Centrifuge at 14,000 X g for 1 minute. Empty the catch tube.
20. Add 500 µl of prepared SEWS-M.
    1. *It is not necessary to re-suspend the pellet during this rinse.*
21. Centrifuge at 14,000 X g for 1 minute. Empty catch tube.
22. Without the addition of any liquid, centrifuge again at 14,000 X g for 2 minutes. *This additional spin removes any excess alcohol that might be present.* Discard the catch tube and place the spin filter in a clean catch tube (*provided in the kit*).
23. Air-dry the spin filter for 5 minutes at room temperature to allow residual ethanol to evaporate.
24. Gently re-suspend the binding matrix in 100 µL DES. (*DES is the molecular-grade water that is provided with the kit*). Incubate at 55°C for 5 min in a heat block.
25. Centrifuge at 14,000 X g for 1 min.

**Removal of PCR Inhibitors**

- Post-DNA Isolation Inhibitor Removal: OneStep™ PCR Inhibitor Removal Kit (Zymo Research; cat no.: D6030; $102 for 50 reactions or 50 soil samples).
  - *Note: The Zymo columns used for removal of the inhibitors contain a wet matrix. Consequently, it may be necessary to spin briefly the columns for 2-3 sec before use if the matrix is adhered to the cap or if most of it is in the upper portion of the tube.*

1. The columns are prepped according to the manufacturer’s instructions: 1) snap off the base; 2) remove the green cap; 3) insert into a collection tube; and 4) spin in a microcentrifuge at exactly 8,000 X g for 3 min.
   1. *If the HRC matrix is dry add 400-600 µl water prior to prepping the column.*
2. Move the prepped column to a 1.5 ml centrifuge tube.
3. Add the DNA suspension from step 25 to the prepped sample column.
4. Centrifuge at 8,000 X g for 1 min.
5. Store the DNA in the freezer (-20°C).

**Quantitative PCR (adapted from Phytopathology 105:220-229)**

- **PCR cocktail:** All samples are analyzed in triplicate (a total of three 20 µl reactions for each soil sample). Data are averaged across the three reactions.

| **Reagent** | **[Initial]** | **Volume/**  **20 µl reaction** | **[Final]** |
| --- | --- | --- | --- |
| ABI SYBR Select MasterMix^a^ | 2X | 10 µl | 1X |
| Forward Primer: Vd-F929-947^b^ | 10 µM | 2 µl | 1 µM |
| Reverse Primer: Vd-R1076-1094^c^ | 10 µM | 2 µl | 1 µM |
| Molecular-grade water | NA | 4 µl | NA |
| DNA sample | NA | 2 µl | NA |

^a^ ABI SYBR Select Mastermix (Thermo Fisher Scientific; cat no.:4472908)

^b^ Forward Primer: Vd-F929-947: 5’-CGTTTCCCGTTACTCTTCT-3’

^c^ Reverse Primer: Vd-R1076-1094: 5’-GGATTTCGGCCCAGAAACT-3’

- **Standard Curve:** A standard curve should be included with every qPCR run.
  1. Extract *V. dahliae* genomic DNA from mycelia or conidia grown in pure culture.
  2. Quantify DNA with the Qubit Fluorometer (ThermoFisher Scientific; cat no.:Q33226) according to the manufacturer’s instructions.
     - *Another fluorometric quantification method can be used, but UV-absorbance-based quantification should not be used.*
  3. Prepare a ten-fold serial dilution of DNA to use as the template for the standard curve.. The DNA concentration should range from 0.5 ng DNA/µl to 0.5 fg DNA/µl
  4. A no template (water) control should also be included with every qPCR run.
- **Thermal Cycler Conditions** (Applied Biosystems StepOnePlus Real-time PCR System; cat no.:4376600)
  1. 10 min @ 95°C
  2. 40 cycles of 30 s @ 95°C, 30 s @ 60°C, and 30 s @ 72°C (measure data).
  3. Melt curve analysis from 60°C, gradually increasing 0.3°C/s to 95°C.
