# Supplementary figures and images for: Evidence of a trans-kingdom plant disease complex between a fungus and plant-parasitic nematodes
Source: PLoS One. 2019 Feb 13;14(2):e0211508. doi: 10.1371/journal.pone.0211508 (PMC6373923; doi:10.1371/journal.pone.0211508)

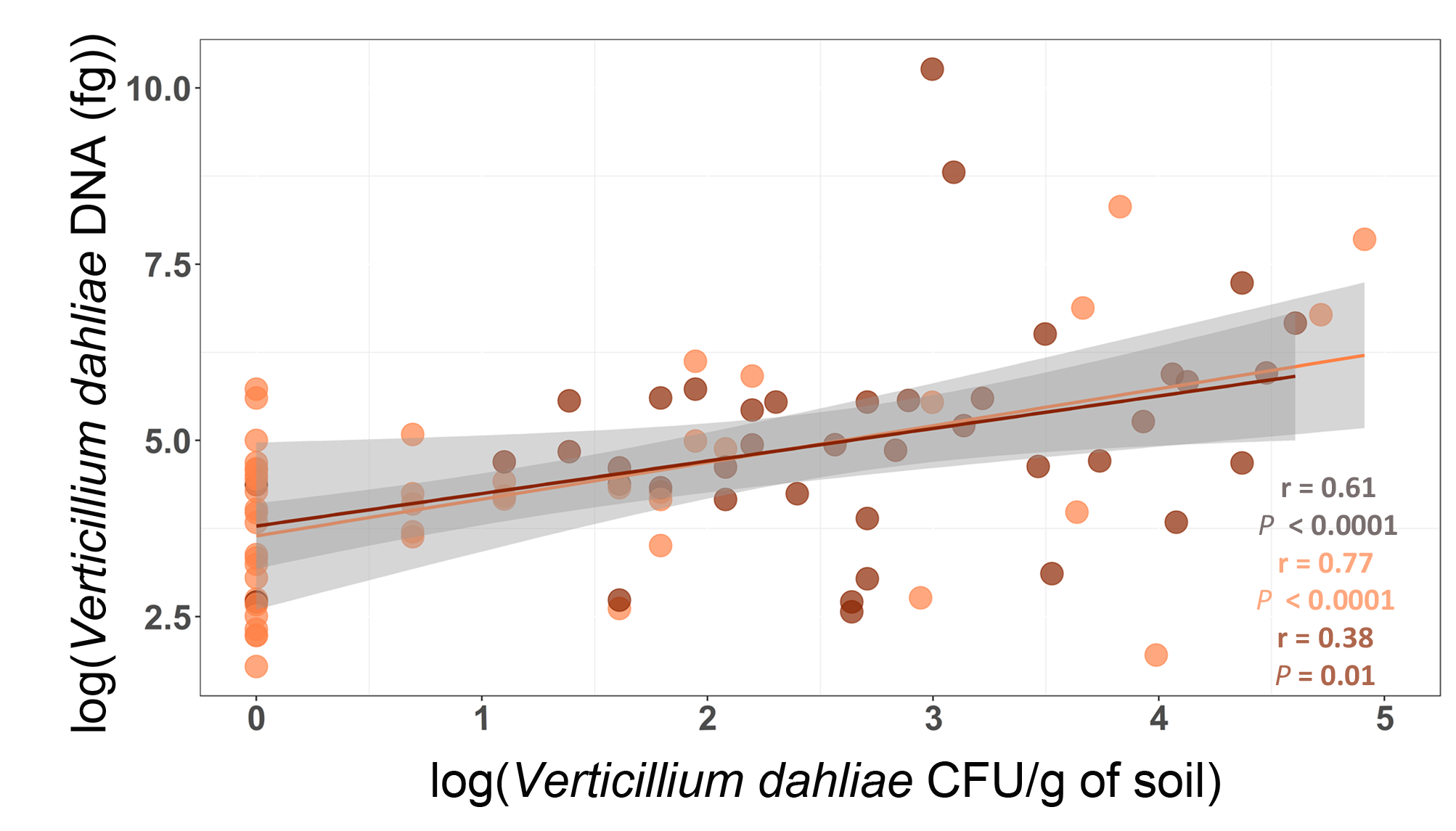

Supplement: S1 Fig — Correlations between estimates of Verticillium dahliae from the quantitative real-time PCR (qPCR) assay and the traditional culture-dependent method. Samples from Washington state are represented in red while samples from Oregon are represented in orange. The correlation coefficient and P-value for all samples are shown in gray while those for samples from Washington and Oregon are shown in red and orange, respectively. (TIF) [file pone.0211508.s002.tif]

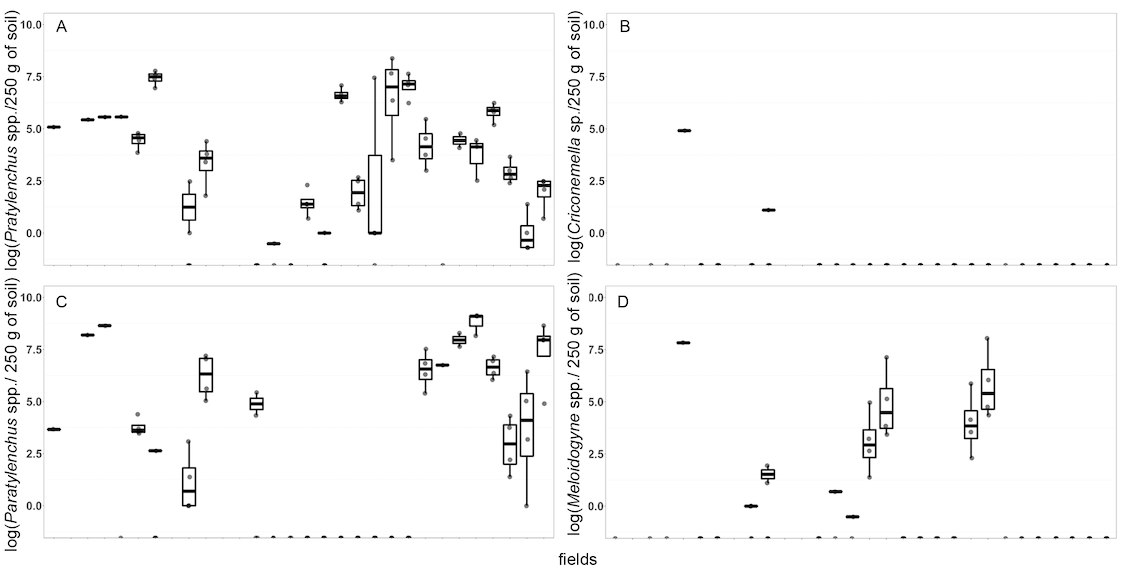

Supplement: S2 Fig — Counts of plant-parasitic nematodes, including Pratylenchus spp. (a), Criconemella sp. (b), Paratylenchus spp. (c), and Meloidogyne spp. (d) are presented for each field. (TIF) [file pone.0211508.s003.tif]

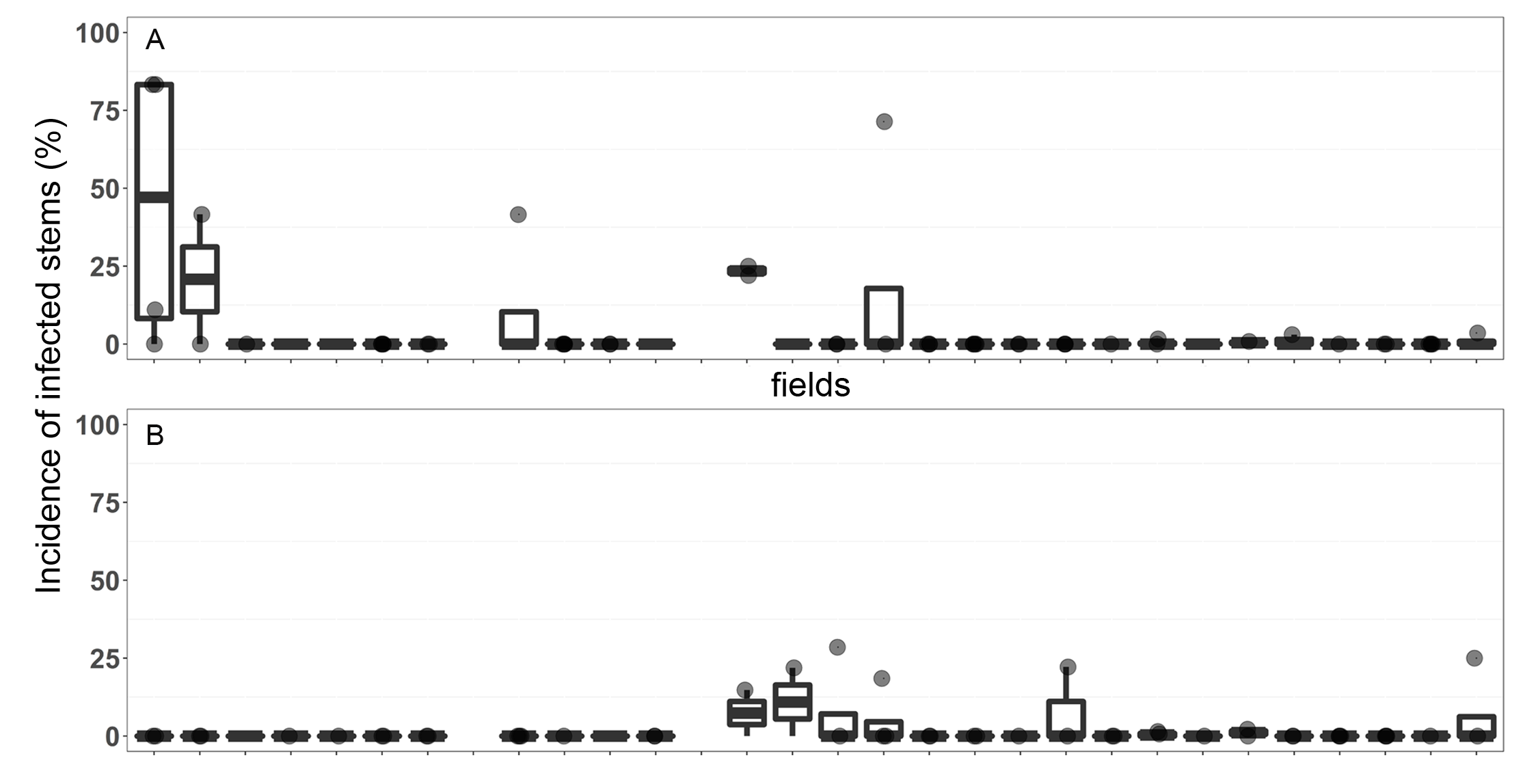

Supplement: S3 Fig — Incidence of susceptible (Mentha gracilis) and resistant (M. spicata) mint stems infected with Verticillium dahliae after a season of growth in desiccated soils collected from commercial mint fields. (TIF) [file pone.0211508.s004.tif]
